# Therapeutic Efficacy of Sirolimus in Skeletal Manifestations of Gorham-Stout Disease in Adults: A Systematic Review

**DOI:** 10.7759/cureus.110745

**Published:** 2026-06-12

**Authors:** Michal Mierzejewski, Sylwia Olizarowicz, Zofia Kania-Bonicka, Michal Majewski, Paulina Szczepanska

**Affiliations:** 1 Faculty of Medicine, Medical University of Warsaw, Warsaw, POL; 2 Department of Dentistry, Masovian Dental Center, Warsaw, POL

**Keywords:** gorham-stout, osteolysis, rapamycin, sirolimus, vanishing bones

## Abstract

Gorham-Stout disease (GSD) is a rare condition characterized by progressive osteolysis and abnormal proliferation of lymphatic vessels. Its pathogenesis involves PI3K/AKT/mTOR pathway hyperactivation, providing a molecular basis for the application of targeted mTOR inhibitors such as sirolimus.

This study aims to systematically review the efficacy and safety of sirolimus in treating adult patients with GSD presenting with skeletal manifestations. A systematic review was conducted in accordance with PRISMA 2020 guidelines using PubMed, Scopus, and EMBASE databases. Data from eligible case reports involving adult patients were extracted and analyzed through a qualitative narrative synthesis. Nine case reports involving 9 patients (mean age 39.3 years) met the inclusion criteria. Patients presented with extensive polyostotic involvement and severe complications such as massive pleural effusions or recurrent chylothorax. Sirolimus therapy yielded a therapeutic response in seven out of nine patients, including two complete and five partial responses. Data from these nine case reports suggest that the intervention may effectively halt active osteolysis and promote the resolution of effusions. While the therapy successfully arrested disease progression and induced significant localized bone formation, complete anatomical reossification of all osteolytic lesions was not achieved in the adult cohort. The treatment was generally well-tolerated, with manageable adverse events (acne, mild mouth sores, hyperlipidemia, nausea, and respiratory infection).

Targeted sirolimus therapy serves as a potential therapeutic option for adult GSD. It necessitates a long-term, multimodal approach, including concurrent anti-resorptive and continuous imaging follow-up. Current evidence is limited by the reliance on heterogeneous case reports. Multicenter prospective registries or international collaborative cohorts are required to validate our results and establish standardized dosing guidelines.

## Introduction and background

Gorham-Stout disease (GSD), also referred to as Gorham-Stout syndrome (GSS) and vanishing bone disease, is a rare osteolytic disorder regarded as a complex lymphatic malformation within the classification framework of the International Society for the Study of Vascular Anomalies. Since its modern clinicopathologic characterization, only a few hundred cases have been reported worldwide, which partly explains the absence of high-level evidence and the lack of standardized management algorithms. Although GSD can occur at any age, it predominantly affects children, adolescents, and young adults, with many patients presenting before the age of 40 years. Reported sex distribution is not entirely uniform across cohorts, but no strong or biologically consistent sex predilection has been established [1-3].

Primarily, GSD is idiopathic, progressive osteolysis involving cortical and medullary bone, usually with minimal compensatory osteogenesis. Consequently, the patient may experience pain, swelling, deformity, pathological fractures, instability, and progressive functional limitation. Disease may be mono- or polyostotic, and the most frequently involved sites include the skull and craniofacial skeleton, clavicle and shoulder girdle, ribs, pelvis or hip, and spine. GSD is not restricted to bone, as peri-osseous soft-tissue infiltration and visceral extension may occur. Thoracic involvement is especially severe. Rib or vertebral disease may lead to pleural effusion or chylothorax through pleural invasion or lymphatic leakage, making respiratory compromise one of the major causes of death. The chronic and unpredictable course of the disease, recurrent pain, mobility restriction and need for prolonged multidisciplinary care together create a major health-related quality-of-life burden [1-4].

Diagnosis remains challenging and is fundamentally based on clinical, radiological and histopathological correlation together with strict exclusion of neoplastic, infectious, metabolic, hereditary and immune-mediated causes of osteolysis. The classic criteria proposed by Heffez and later modified in the literature remain highly practical in this setting. Histologically, lesions typically show nonmalignant proliferation and dilation of thin-walled lymphatic or vascular channels without cellular atypia, often accompanied by positive lymphatic markers such as PROX1 and D2-40. Imaging is important throughout the diagnostic pathway. Plain radiographs and computed tomography (CT) generally demonstrate progressive osteolysis, cortical destruction, medullary resorption, little or no osteogenic reaction, and usually lack of osteosclerosis or periosteal reaction. Magnetic resonance imaging (MRI) is valuable for detecting early marrow signal abnormalities and defining adjacent soft-tissue and organ involvement [1,3-5].

Current concepts of pathogenesis suggest that bone may not function as the primary target organ, pointing instead to a more systemic involvement. Osteolysis may be secondary to pathological lymphatic endothelial proliferation and its interaction with the bone microenvironment. Experimental work has shown that lymphatic endothelial cells can amplify RANKL-mediated osteoclastogenesis and bone resorption through macrophage colony-stimulating factor, while VEGF-C-driven lymphangiogenesis within bone can reproduce a GSD-like osteolytic phenotype in murine models. This lymphatic-bone crosstalk is driven by local osteoclastogenic factors, such as IL-6 and RANKL, within the lesion. Molecular studies also support biologic heterogeneity converging on signaling pathways: somatic activating KRAS variants have been identified in affected GSD tissue, KRAS-driven lymphatic endothelial models recapitulate bone lymphatic invasion and chylothorax, and broader genomic analyses suggest additional abnormalities involving PTEN/PI3K-axis regulators and recurrent fusion events. Importantly, while recurrent PIK3CA mutations are better established in related lymphatic anomalies such as generalized lymphatic anomaly than in GSD itself, the available evidence strongly supports the relevance of the PI3K/AKT/mTOR axis across this disease spectrum [2,6-13].

Because GSD is a rare disorder, no universally accepted treatment standard exists. In clinical practice, management is individualized and often stepwise. Initial treatment generally focuses on conservative measures and bone-directed antiresorptive therapy, most commonly bisphosphonates and, in more selective case-based use, denosumab or other anti-osteoclast approaches, with the aim of reducing osteoclastic resorption and pain. Progression threatening structural integrity may require surgery for biopsy, resection, decompression, stabilization, or reconstruction. Radiotherapy can achieve local control in selected symptomatic or unresectable lesions, typically in the 30-45 Gy range, but its long-term toxicity seems to remain relevant, particularly in younger patients. A major limitation of these first- and second-line approaches is that they largely treat the skeletal consequence of GSD rather than its primary lymphatic driver, which helps explain treatment refractoriness and relapse in multifocal and thoracic disease [1,3,14,15].

Against this background, mTOR inhibition provides a compelling translational rationale. The PI3K/AKT/mTOR axis is a common pathway for pathological lymphangiogenesis, and sirolimus (rapamycin) can suppress abnormal lymphatic endothelial growth in pathway-driven lymphatic disorders. In parallel, mTOR signaling intersects directly with osteoclast differentiation and bone resorption biology, suggesting that sirolimus may act both upstream on lymphatic proliferation and, at least in part, at the level of bone remodeling, even if preclinical data indicate that these osteoclast effects may be context- and dose-dependent. Clinically, sirolimus is already established as a systemic option in complicated vascular anomalies and has shown activity in mixed cohorts, where symptoms and functional status frequently improve, and osseous disease often stabilizes. However, the available evidence remains fragmented and frequently pooled across pediatric and adult populations or across different complex lymphatic anomalies. Consequently, the long-term clinical and radiological effects of sirolimus, specifically on bony manifestations in adults with GSD, have not been synthesized. [13,16-20].

This systematic review aims to evaluate the efficacy and safety of targeted sirolimus therapy in adult patients with skeletal manifestations of Gorham-Stout disease.

## Review

Materials and methods

**Figure 1:**
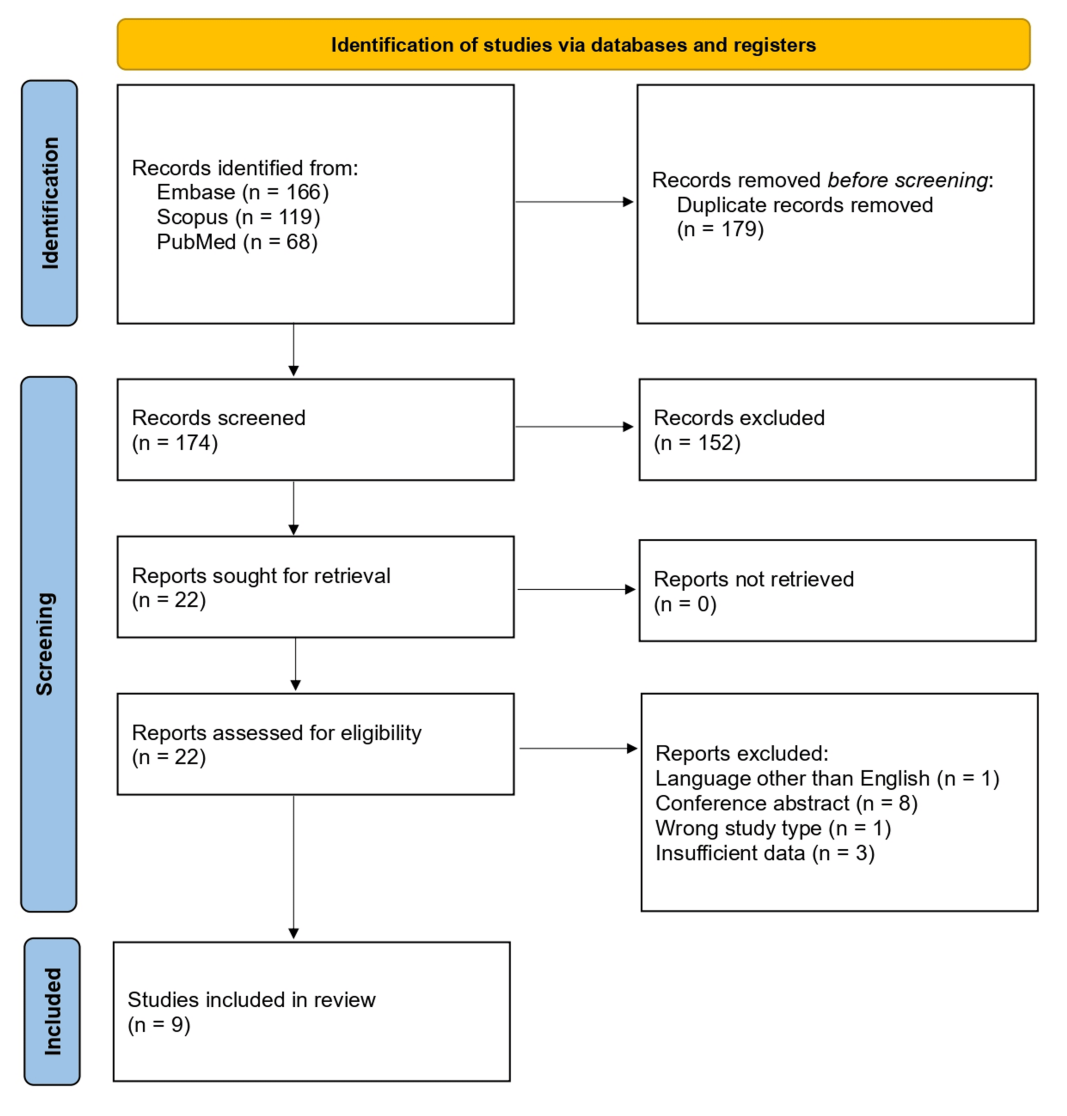
PRISMA 2020 flow diagram depicting the selection process of studies included in the review

This systematic review was conducted in accordance with the Preferred Reporting Items for Systematic Reviews and Meta-Analyses (PRISMA) 2020 guidelines [21]. A comprehensive database search was performed on April 14, 2026, using PubMed, Scopus, and EMBASE databases. The search query ("Sirolimus" OR "Rapamycin") AND ("Gorham-Stout" OR "GSS" OR "GSD") yielded 353 results, of which 179 were duplicates. The complete search strategy is provided in Appendix 1. Screening titles and abstracts revealed 22 eligible studies, of which all were later retrieved. Nine studies met the final inclusion criteria for data review. The PRISMA flow chart is presented in Figure 1. All screening steps were undertaken independently by four reviewers, with disagreements resolved by consensus. The PICO (Population, Intervention, Comparator, Outcome) framework defined the population as adult (age ≥ 18 years old) patients diagnosed with Gorham-Stout disease with skeletal manifestations treated with sirolimus. Studies not focusing on skeletal manifestations were excluded, as well as studies on pediatric and mixed pediatric/adult populations. Review articles, conference abstracts, and studies not providing sufficient data or in a language other than English were also excluded. The methodological quality of the included case reports was appraised using the Joanna Briggs Institute (JBI) Critical Appraisal Tool [22]. The risk of bias assessment is presented in Appendix 2.

Extracted data consisted of study characteristics (author, year, country, design), patient demographics (sex, age, age of onset, age at sirolimus initiation), intervention specifics (dose of sirolimus, concentration in blood serum, duration of treatment, concurrent treatment), and clinical outcomes (adverse effects, radiological findings, efficacy, recurrence). Clinical outcomes following sirolimus therapy were stratified into complete response (CR), partial response (PR), and suboptimal response (SR). CR was defined as the achievement of clinical and radiological remission, characterized by the complete remission of disease-related symptoms, such as osteodynia, dyspnea, and chylothorax, alongside the remission of osteolysis in radiological examination. Although inactive residual fibrosis or permanent structural bone deformities were irreversible, classification of CR required absolute independence from symptomatic and supportive interventions (e.g., pleural drainage) without disease relapse. PR was defined as a clinically meaningful improvement that did not meet the criteria for complete response. This category included patients demonstrating a significant reduction in symptom severity, such as decreased pleural effusion volume or reduced pain severity, but with persistent clinical disease activity or incomplete radiological stabilization. Patients requiring ongoing supportive treatment, repeated drainage procedures, or analgesia to suppress symptoms were strictly categorized as PR. Suboptimal response was defined as a lack of therapeutic benefit, characterized by unremitting or progressive osteolytic disease, exacerbation of life-threatening complications (e.g., refractory chylothorax, respiratory failure), or the necessity to transition to alternative systemic therapies or palliative care due to primary treatment failure or severe clinical deterioration. To ensure an accurate classification of treatment outcomes, the initial predefined response categories were adjusted after data extraction to include irreversible bone changes. Because of the lack of control groups and heterogeneity of the included case reports, a meta-analysis was not feasible. The efficacy of sirolimus was analyzed with a qualitative narrative synthesis.

Results

A total of nine case reports describing nine patients (five males, four females) met the inclusion criteria and were analyzed in this review [23-31]. The mean age of the cohort at the time of sirolimus initiation was 39.3 years, with a range from 19 to 78 years. No genetic testing was reported in the original reports to identify potential pathogenic mutations in any of the analyzed cases. Patients presented extensive polyostotic involvement, frequently affecting the ribs, spine, skull, and pelvis. Massive pleural effusion or recurrent chylothorax was a common complication observed in the majority of the cohort [23-25,28-30]. Several patients initiated sirolimus therapy due to refractory disease following the failure of prior systemic interventions, including zoledronic acid and pegylated interferon alpha 2b [24,27]. Sirolimus was administered orally, with initial daily dosages typically ranging from 1 mg to 2 mg [23,26,27,29,30]. Dose adjustments were required in specific cases, including titration up to 4 mg daily or up to 4 mg twice a day (yielding a total daily dose of 8 mg) to achieve therapeutic targets [23,24], or the administration of a single lead dose of 4 mg followed by a maintenance dose of 1.5 mg [25]. The target sirolimus plasma concentrations varied between patients because there are currently no standardized management guidelines for Gorham-Stout disease, requiring individualized dosage titration based on clinical severity, patient tolerance, and therapeutic response. Target trough blood concentrations were closely monitored and generally maintained between 4 ng/mL and 15 ng/mL to optimize efficacy while minimizing toxicity [23-26,28,30]. Concomitant therapies were utilized in several cases, most notably intravenous zoledronic acid and vitamin D supplementation [24,26,29,30]. Treatment duration varied widely across the analyzed studies, extending from three months to over three years [26,28]. A therapeutic response was achieved in seven out of nine patients, comprising two complete clinical responses characterized by clinical remission and prominent, though localized, maxilla reossification, and five partial responses showing improvement in lytic lesions and resolution of effusions [23-27,29,30]. Two patients experienced a suboptimal response to the intervention [28,31]. The pharmacological intervention was generally tolerated, though specific adverse events were documented, including acne, mild mouth sores, hyperlipidemia, nausea, and respiratory infection [23,24,26,30]. One female patient experienced metrorrhagia, necessitating a temporary interruption of the treatment, which was successfully restarted without further complications [25]. Furthermore, disease recurrence was explicitly noted in two cases following the planned discontinuation or temporary suspension of sirolimus, suggesting a potential necessity for prolonged maintenance therapy to sustain clinical benefits [24,25]. 

Radiological evaluations before sirolimus initiation revealed an extensive spectrum of osteolytic lesions across the cohort. The axial skeleton and craniofacial bones were predominantly affected, with massive resorption observed in the maxilla, pterygoid process, orbit, temporal bone, and mandible [26-28]. Costal and vertebral involvement was frequently documented, presenting as multiple lytic lesions, bone defects, and associated pathologic fractures [23-25,29,30]. Furthermore, osteolysis extended to the appendicular skeleton and pelvic girdle, involving the scapulae, iliac bones, sacrum, and femurs [23,29,31]. These skeletal manifestations were strongly associated with severe extraskeletal complications, most notably massive unilateral or bilateral pleural effusions and recurrent chylothorax [23-25,28-30]. Additional rare radiological findings included ascites and complex neurological manifestations such as tonsillar herniation, syringomyelia, and cerebrospinal fluid leaks secondary to structural deterioration [29,31]. Following sirolimus administration, follow-up imaging in patients achieving a therapeutic response demonstrated marked radiological improvements. These changes were characterized by the arrest of osteolysis, prominent reossification and mineralization of previously affected bones, such as the maxilla and ribs, and the complete or partial resolution of pleural effusions [23-26]. Studies included in the review are presented in Table 1.

**Table 1:** Summary of the characteristics of the selected studies NG - Not Given, IU - International Units, IV - Intravenous

Study	Year	Country	Study design	Gender	Age of onset	Age	Dose of sirolimus	Sirolimus blood concentration	Duration of treatment	Concurrent treatment	Adverse effects	Efficacy	Recurrence
Amezquita et al. [23]	2024	Colombia	Case report	Male	NG	19	Initiated at 2 mg/day, adjusted to 4 mg/day, later reduced to 2 mg/day	Target: 5 ng/ml, Actual: 5 ng/ml	12 months	NG	Acne	Partial response	None reported during 1-year follow-up
Cramer et al. [24]	2016	USA	Case report	Male	18	18	1 mg/day, titrated to 4 mg/day	Target: 9-12 ng/ml	18 months	Zoledronic acid 4 mg IV monthly	Mild mouth sores, hyperlipidemia	Partial response	Yes, 3 months after discontinuing therapy
García et al. [25]	2016	Spain	Case report	Female	42	43	Single lead dose of 4 mg, followed by 1.5 mg/day	Target: 4-10 ng/ml, Actual: 3.8-4.3 ng/ml	10 months	NG	Metrorrhagia	Complete response	Yes, following temporary suspension of sirolimus (resolved upon reintroduction)
Gvetadze et al. [26]	2025	Russia	Case report	Male	39	40	2 mg/day, reduced to 1 mg/day	Target: 6 ng/ml, Actual: 11.9 ng/ml initially, then 4.5-5.0 ng/ml	36 months	Zoledronic acid 4 mg every 4 weeks, vitamin D 5000 IU once daily	Nausea	Complete response	None
Forero Saldarriaga et al. [27]	2021	Colombia	Case report	Male	30	33	2 mg/day	NG	~18 months	None	None	Partial response	None
Thompson et al. [28]	2021	USA	Case report	Female	60	60	NG	Target: 7-10 ng/ml, then 10-15 ng/ml	3 months	Bilateral chest tubes, mechanical ventilation	NG	Suboptimal response	NG
Wojciechowska-Durczynska et al. [29]	2022	Poland	Case report	Female	42	42	2 mg/day	NG	9 months	Zoledronic acid 4 mg IV (single dose), opioid analgesics	NG	Partial response	NG
Yamaki et al. [30]	2025	Japan	Case report	Male	78	78	2 mg/day	Target: 5-15 ng/mL	~18 months	Vitamin D supplementation	Respiratory infection (Klebsiella pneumoniae)	Partial response	None
Yoshimoto et al. [31]	2018	Japan	Case report	Female	2	21	NG	NG	11 months	NG	NG	Suboptimal response	NG

Discussion

Pathogenesis and Mechanism of Action

The reason for using targeted sirolimus therapy in refractory GSD is supported by systemic efficacy in other conditions driven by PI3K/AKT/mTOR pathway dysregulation. Recent genomic studies showed the role of somatic mutations in GSD. Activating somatic mutations in the KRAS gene have been identified in tissue specimens of patients with GSD, including the p.Q61R and p.G12V variants [9,10]. These mutations lead to the constitutive activation of the RAS/MAPK and PI3K/AKT/mTOR signaling pathways, which promote pathological cell proliferation [9]. While current data suggest this hypothesis, these observations may not be universally applicable across the entire GSD patient population due to potential clinical heterogeneity. Significance of KRAS pathway hyperactivity has been confirmed in mouse models, where the expression of the mutated protein induced the formation of ectopic lymphatic vessels in bones and impaired the development of lymphatic valves. These changes were suppressed following the administration of trametinib, a MEK1/2 inhibitor [10]. In addition to point mutations, analyses have demonstrated that GSD can also be associated with structural DNA rearrangements. Aberrations have been identified in chromosomes 7, 12, and 20, including a gene fusion involving ATG101, which may disrupt macroautophagy processes [12]. Whether driven by activating somatic mutations or structural gene rearrangements, these pathogenetic mechanisms may lead to the hyperactivation of the PI3K/AKT/mTOR axis. This suggests a molecular rationale for the application of targeted mTOR inhibitors, such as sirolimus, to suppress pathological cell proliferation in GSD.

A 12-month targeted sirolimus therapy in patients with tuberous sclerosis complex or sporadic lymphangioleiomyomatosis significantly reduced the mean angiomyolipoma volume to 53.2% of the baseline value. Furthermore, suppressing the overactive mTOR signaling pathway yielded functional pulmonary improvements in the lymphangioleiomyomatosis cohort, notably demonstrating a mean increase in forced vital capacity of 390 ml [32]. A phase II trial established the efficacy and safety of targeting the PI3K/AKT/mTOR pathway with oral sirolimus in patients with vascular anomalies, including microcystic lymphatic malformations. After 12 courses, 85% of patients demonstrated a partial response, evidenced by improvements in quality of life and radiologic parameters, with well-tolerated trough levels of 10 to 15 ng/mL [16].

**Figure 2:**
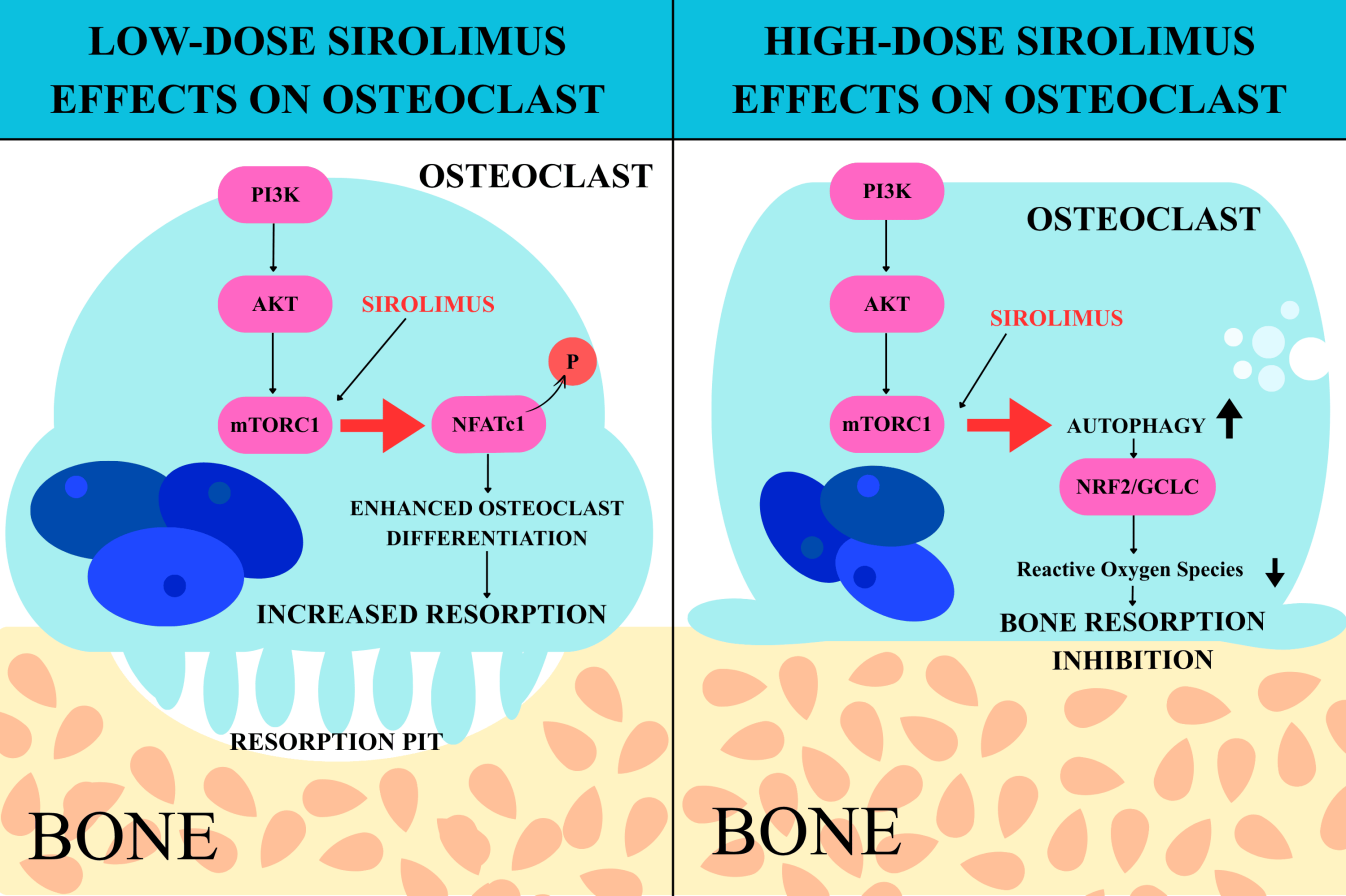
Effect of high and low dose sirolimus on osteoclasts The illustrated molecular pathways and cellular mechanisms are based on findings from preclinical animal and in vitro laboratory models. This figure was created using Canva (Canva Pty Ltd., Sydney, Australia).

The effects of sirolimus on bone metabolism appear to be dose-dependent. In preclinical animal and laboratory models, high concentrations inhibit osteoclastogenesis and protect against inflammatory bone loss by suppressing oxidative stress via the Nrf2/GCLC pathway and increasing autophagic flux [20]. Experimental models suggest that low, clinically relevant doses can paradoxically exacerbate osteoclast differentiation and bone resorption. This dual regulation occurs because mTORC1 acts as a switch from proliferation to differentiation. Its inhibition by sirolimus reduces NFATc1 phosphorylation, leading to the subsequent activation of osteoclastogenic transcription factors [19]. While sirolimus is utilized to target the PI3K/AKT/mTOR axis for its antiangiogenic properties in osteolytic conditions like GSD, its efficacy in completely stopping bone resorption remains limited [9]. The effect of sirolimus on bones is shown in Figure 2. 

Radiological Evaluation

The radiological phenotype of GSD is characterized by progressive osteolysis, which manifests on radiographs as lytic lesions frequently involving the spine and pelvis, often resulting in complications such as scoliotic curvature and pathological fractures. Osteolysis may be caused by the abnormal proliferation of lymphatic vessels that reflux directly into the bone marrow cavity. CT identifies associated non-osseous manifestations, presenting as blurry, infiltrative soft-tissue spreading around the affected bone. Dynamic contrast-enhanced magnetic resonance lymphangiography visualizes abnormal collateral lymphatic vessels infiltrating both the intraosseous lesions and the surrounding peri-osseous soft tissues [4]. Tracking those imaging parameters may help evaluate the efficacy of the therapy. The radiological evolution of GSD during sirolimus treatment is characterized by the definitive cessation of active osteolysis. Continuous CT surveillance serves as an indicator of treatment response, demonstrating the transition of the active lesion into a chronic, stable phase as well as local fibrosis and scarring at the initial osseous defect [26]. While CT shows structural changes, MRI may be preferred for monitoring the evolution of radiological changes. Comparative analysis of T2-weighted sequences before and during treatment documents response indicators such as a significant reduction in the fluid volume of pathological lymphatic spaces. For intraosseous lytic lesions, complete signal resolution on MRI is delayed compared to soft tissues, largely due to the limited blood supply of bone tissues [17]. Furthermore, a recent study evaluating sirolimus therapy in patients with rare lymphatic anomalies demonstrated that while partial volumetric reduction of target lesions is achievable, complete radiological resolution may not occur. Moreover, the study highlighted a clear clinical-radiological dissociation, noting that the significance of radiological lesion reduction does not strictly correlate with the degree of symptomatic improvement. Even in cases classified radiologically as stable disease without significant volumetric shrinkage, patients frequently experienced improvements in severe clinical complications such as pain or effusions [18]. However, despite clinical stabilization and cessation of progressive bone loss, radiological imaging may reveal a persistent structural defect with an absence of spontaneous reossification.

The explanation for this lack of structural bone healing might be found at the molecular level. Bone repair requires the differentiation of mesenchymal stem cells into mature osteoblasts. IGF-1 drives this differentiation specifically through the activation of the mTOR signaling pathway, demonstrating that pharmacological mTOR inhibition directly stops osteoblast maturation and bone mineralization. Because the physiological concentration of IGF-1 within the bone matrix declines with age, we hypothesize that adult patients might lack the anabolic stimulus required to overcome this therapeutic mTOR blockade. This age-related decline could speculatively account for the persistent lack of structural reossification [33]. However, this limitation may appear to be age-dependent. Unlike adults, pediatric patients could retain a growth stimulus capable of overriding this blockade. A recent case report demonstrated that oral sirolimus successfully halted progressive osteolysis and promoted secondary ossification in a pediatric patient with Gorham-Stout disease [34]. By inhibiting the hyperactive mTOR pathway, the treatment led to sustained clinical and radiological improvement.

Clinical Management

Historically, conventional medical treatments for complex lymphatic anomalies like GSD, including steroids, interferon, and chemotherapeutic agents, have been limited and produced highly variable clinical outcomes. In contrast, targeted therapy with sirolimus offers a mechanism-driven approach that has been shown to effectively stabilize the disease and may prevent the progression of bone lesions. However, while sirolimus directly suppresses the primary lymphatic driver, clinical evidence suggests that monotherapy may not be sufficient for optimal management of the severe osseous manifestations of GSD. The addition of sirolimus to a concomitant anti-resorptive treatment, such as bisphosphonate therapy with zoledronic acid, has been documented to provide clinical benefits, leading to further improvements in bone symptoms and overall patient quality of life. Consequently, the modern clinical management of GSD requires a multimodal pharmacological approach to simultaneously suppress pathological neo-lymphangiogenesis and massive osteoclastogenesis. Unfortunately, it requires long-term management and lacks a definitive cure [17].

The management of skeletal manifestations in adult patients with GSD may require a coordinated pharmacological strategy centered on targeted anti-angiogenic therapy and bone matrix protection. Treatment is usually initiated with oral sirolimus at a dose of 1-2 mg/day. During the aggressive phase of the disease, the dose is typically adjusted with target concentrations (Cmin) between 4 and 15 ng/mL. Once clinical and radiographic stabilization is achieved, the regimen might be transitioned to a maintenance phase with a reduced Cmin target of 2-6 ng/mL [23-31]. The concurrent and continuous administration of anti-resorptive agents, such as intravenous zoledronic acid or denosumab, is suggested [1,17,23-31].

Safety

Sirolimus is contraindicated in patients with hypersensitivity to the drug or its components. Primary clinical restrictions include active systemic infections, severe hepatic impairment, and significant cytopenia. Furthermore, its use is prohibited during the perioperative period due to impaired angiogenesis and wound healing [1,35]. Although data specific to GSD are limited, evidence from broader literature on complex vascular anomalies and solid organ transplantation indicates that the adverse effect profile of sirolimus is not rare. In these larger cohorts, approximately 80% of patients experience at least one side effect during therapy. Hematologic toxicities, such as neutropenia and anemia, occur in roughly 27% of cases. Immunological risks have been reported in up to 37% of patients. Common mucosal and gastrointestinal manifestations include stomatitis and mouth sores, which affect approximately 30% of the treated population. Additionally, patients may experience dermatological reactions, such as acne, gastrointestinal distress like nausea, menstrual irregularities, such as metrorrhagia, and metabolic complications, such as hyperlipidemia and hypertriglyceridemia. Although these effects are common, they are generally considered manageable through dose adjustment and therapeutic drug monitoring [35,36].

Limitations

This review is primarily limited by its reliance on case reports (n = nine studies, nine patients). The small sample size is a direct result of the condition's rarity and the limited number of published cases, rather than strict inclusion criteria. Furthermore, focusing exclusively on the adult population further limited the number of eligible studies, as those involving the pediatric population were excluded. Consequently, this reliance on case reports introduces selection bias and precludes the performance of a quantitative meta-analysis to calculate precise efficacy rates. Additionally, the absence of control groups, combined with the use of concurrent therapies such as zoledronic acid and denosumab in many cases, complicates the assessment of sirolimus’s independent therapeutic effects. Given the rarity of the disease, prospective randomized controlled trials are challenging to implement. Instead, multicenter prospective registries or international collaborative cohorts are required to validate our results and establish standardized dosing guidelines.

## Conclusions

Targeted sirolimus therapy serves as a potential therapeutic option for the management of GSD with skeletal manifestations in adult patients. While it might provide clinical stabilization, complete reossification is frequently absent in the adult population. Consequently, sirolimus requires a long-term and multimodal approach, necessitating concurrent administration of anti-resorptive agents, such as zoledronic acid or denosumab, during the maintenance phase. It is generally tolerated, with adverse effects considered manageable through therapeutic drug monitoring and dose adjustments. The interpretation of these outcomes requires caution due to methodological constraints. Due to the current reliance on a small number of heterogeneous case reports lacking control groups, these outcomes must be interpreted with caution. Finally, multicenter prospective registries or international collaborative cohorts are needed to validate these findings and standardize dosing regimens for GSD.

## Appendices

Appendix 1: Complete search strategy for each database

PUBMED

("Sirolimus" OR "Rapamycin") AND ("Gorham-Stout" OR "GSS" OR "GSD")

SCOPUS

TITLE-ABS-KEY ("Sirolimus" OR "Rapamycin") AND TITLE-ABS-KEY ("Gorham-Stout" OR "GSS" OR "GSD")

EMBASE

("Sirolimus" OR "Rapamycin") AND ("Gorham-Stout" OR "GSS" OR "GSD")

Appendix 2: Risk of bias assessment using the Joanna Briggs Institute (JBI) Critical Appraisal Tool for case reports

**Table 2:** Risk of bias assessment using Joanna Briggs Institute (JBI) Critical Appraisal Tool for case reports Q1 – Q8 indicate questions 1 to 8 based on the JBI Risk Assessment Tool for case reports [22]. The risk of bias was considered high if the study achieved up to 50% of 'yes' scores, moderate if it scored between 51% and 75% of 'yes' scores, and low with more than 75% of 'yes' scores.

Study	Q1	Q2	Q3	Q4	Q5	Q6	Q7	Q8	Sum of Yes	Risk of Bias Level
Amezquita et al. [23]	Yes	Yes	Yes	Yes	Yes	Yes	Yes	Yes	8	Low
Cramer et al. [24]	Yes	No	No	Yes	Yes	Yes	Yes	Yes	6	Moderate
Garcia et al. [25]	Yes	Yes	Yes	Yes	Yes	Yes	Yes	Yes	8	Low
Gvetadze et al. [26]	Yes	Yes	Yes	Yes	Yes	Yes	Yes	Yes	8	Low
Forero Saldarriaga et al. [27]	Yes	Yes	No	Yes	No	No	No	Yes	4	High
Thompson et al. [28]	Yes	No	Yes	Yes	Yes	Yes	No	Yes	6	Moderate
Wojciechowska-Durczynska et al. [29]	Yes	Yes	No	Yes	Yes	Yes	No	Yes	6	Moderate
Yamaki et al. [30]	Yes	Yes	Yes	Yes	Yes	Yes	Yes	Yes	8	Low
Yoshimoto et al. [31]	Yes	Yes	Yes	Yes	No	Yes	No	Yes	6	Moderate
